# Pomalidomide Shows Significant Therapeutic Activity against CNS Lymphoma with a Major Impact on the Tumor Microenvironment in Murine Models

**DOI:** 10.1371/journal.pone.0071754

**Published:** 2013-08-05

**Authors:** Zhimin Li, Yushi Qiu, David Personett, Peng Huang, Brandy Edenfield, Jason Katz, Darius Babusis, Yang Tang, Michael A. Shirely, Mehran F. Moghaddam, John A. Copland, Han W. Tun

**Affiliations:** 1 Department of Cancer Biology, Mayo Clinic, Jacksonville, Florida, United States of America; 2 Department of Drug Metabolism and Pharmacokinetics, Celgene Corporation, San Diego, California, United States of America; 3 Department of Hematology/Oncology, Mayo Clinic, Jacksonville, Florida, United States of America; University of Bari Medical School, Italy

## Abstract

Primary CNS lymphoma carries a poor prognosis. Novel therapeutic agents are urgently needed. Pomalidomide (POM) is a novel immunomodulatory drug with anti-lymphoma activity. CNS pharmacokinetic analysis was performed in rats to assess the CNS penetration of POM. Preclinical evaluation of POM was performed in two murine models to assess its therapeutic activity against CNS lymphoma. The impact of POM on the CNS lymphoma immune microenvironment was evaluated by immunohistochemistry and immunofluorescence. In vitro cell culture experiments were carried out to further investigate the impact of POM on the biology of macrophages. POM crosses the blood brain barrier with CNS penetration of ~ 39%. Preclinical evaluations showed that it had significant therapeutic activity against CNS lymphoma with significant reduction in tumor growth rate and prolongation of survival, that it had a major impact on the tumor microenvironment with an increase in macrophages and natural killer cells, and that it decreased M2-polarized tumor-associated macrophages and increased M1-polarized macrophages when macrophages were evaluated based on polarization status. In vitro studies using various macrophage models showed that POM converted the polarization status of IL4-stimulated macrophages from M2 to M1, that M2 to M1 conversion by POM in the polarization status of lymphoma-associated macrophages is dependent on the presence of NK cells, that POM induced M2 to M1 conversion in the polarization of macrophages by inactivating STAT6 signaling and activating STAT1 signaling, and that POM functionally increased the phagocytic activity of macrophages. Based on our findings, POM is a promising therapeutic agent for CNS lymphoma with excellent CNS penetration, significant preclinical therapeutic activity, and a major impact on the tumor microenvironment. It can induce significant biological changes in tumor-associated macrophages, which likely play a major role in its therapeutic activity against CNS lymphoma. POM should be further evaluated in clinical trials.

## Introduction

Primary central nervous system lymphoma (PCNSL) is most frequently a diffuse large B cell lymphoma (DLBCL) confined to the CNS and carries a poor prognosis [1]. The CNS tumor microenvironment plays an important role in the biology of CNS lymphoma. The standard therapy consists of high-dose methotrexate and high-dose cytidine arabinoside (ara-c) with or without radiation. Although there has been an improvement in the survival due to these treatments, the prognosis of CNS lymphoma remains poor compared to systemic DLBCL [1]. Current therapeutic agents target lymphoma cells and have no significant impact on the tumor microenvironment. In addition, the blood brain barrier is a major obstacle for effective treatment of CNS lymphoma. As such, therapeutic agents with better efficacy, excellent CNS penetration, and impact on the tumor microenvironment as well as lymphoma cells need to be developed.

Pomalidomide, a thalidomide analogue and a novel immunomodulatory agent, has shown in vitro activity against lymphoma cell lines and in vivo pre-clinical activity against systemic lymphoma in a murine model [2,3]. Lenalidomide, another thalidomide analogue with immunomodulatory activity, has shown therapeutic activity against the activated B cell subtype of systemic diffuse large B cell lymphoma [4], which is the subtype of DLBCL seen in more than 95% of PCNSL [5]. Case reports have also indicated activity of lenalidomide in refractory intra-ocular lymphoma [6], and blastoid mantle cell lymphoma affecting the CNS [7].

Herein, we reported the findings from our comprehensive preclinical evaluation of POM for therapeutic use against CNS lymphoma. In this study, we performed CNS pharmacokinetics of POM in rats, preclinical evaluation of POM in two murine CNS lymphoma models, and in-depth analysis of the impact of POM on the tumor immune microenvironment with an emphasis on tumor-associated macrophages. The results indicate that POM is a promising agent for CNS lymphoma with excellent CNS penetration, significant preclinical therapeutic activity, and a major impact on tumor microenvironment. As such, we have generated a preclinical dataset, which will help exploration of the role of POM in management of CNS lymphoma in clinical trials.

## Materials and Methods

CNS pharmacokinetic analysis of POM was performed in a Celgene laboratory. The Tun laboratory at Mayo Clinic, Florida performed all the other experiments.

### 1) CNS pharmacokinetic analysis of pomalidomide

#### Drugs

Compounds CC-4047 (pomalidomide, MW 273.25, C_13_H_11_N_3_O_4_) and CC-6032 (MW 287.27, C_14_H_13_N_3_O_4_) from Celgene were used in pharmacokinetic analyses. CC-6032 was used as the probe calibrator in the microdialysis experiment.

#### Microdialysis

A total of 3 male CD-IGS rats were used. Stomach-cannulated CD-IGS rats (male, weight range: 250–300 g) supplied by Charles River Laboratories (Wilmington, MA) were used in this study. Following surgery, all animals were housed in BASi Raturn® (West Lafayette, IN) containment systems with standard bedding material. Rat chow and water were available ad libitum, and all animals were kept in an ambient temperature room under a 6 am to 6 pm 12-hour lighting schedule.

Animal surgeries consisted of implanting a CMA/20 14/10PC vascular microdialysis probe (Part # 8309571, CMA Microdialysis, North Chelmsford, MA) in the jugular vein, according to an IACUC protocol. Each animal was then stereotaxically implanted with an intracerebral guide directed toward the top of the striatum (A/P: 0.7, L/M: -3.0, D/V: -3.0; from bregma), according to a rat stereotaxic atlas [8]. A BASi BR-4 brain microdialysis probe (Part # MD-2204, BASi, West Lafayette, IN) was inserted prior to recovery, and the probes were slowly perfused (0.5 µL/min) with either sterile lactated Ringer’s (brain) or Dulbecco’s phosphate-buffered saline (D-PBS; blood). Animals were allowed to recover for at least 24 hours prior to dosing. On the day of dosing, the blank perfusate was replaced with perfusate containing the probe calibrator, and flow was set to1.25 µl/min. CC-4047 was administered as a single PO administration via the stomach cannula, at 50 mg/kg (5 mL/kg) in a 0.5% carboxymethylcellulose / 0.25% Tween® 80 suspension formulation. Microdialysate was collected in a cooling fraction collector, set at 4^o^C (Eicom # EFC-82, EFR-82, Eicom, San Diego, CA) at intervals of 25 minutes for 10 hours after dosing. To calculate area-under-the-curve (AUC), the corrected concentration of each sample was multiplied by the interval over which the sample was collected; in this case 25 minutes, and divided by 60 minutes per hour. The sum of these values represented the total AUC value over the specified time range. To generate graphs, the concentration at each time point was plotted at the mid-point of each collection interval. Microdialysates were collected at the specified time points and analyzed for CC-4047 concentration using a liquid chromatography-tandem mass spectrometry (LC-MS/MS) assay, within 12 hours. Interim storage was at 4° C.

#### Mass Spectral Analysis

Microdialysate samples were injected directly into the LC-MS/MS system without processing. Chromatographic separation was achieved using a Phenomenex Synergi C_18_ column (50 x 4.6 mm, 4 µm) with gradient elution of 0.1% formic acid in water and 0.1% formic acid in acetonitrile. Detection and quantitation were performed using positive electrospray in multiple reaction-monitoring (MRM) modes on a Waters Micromass Ultima tandem mass spectrometer. Transition ions monitored were m/z 274.1 to m/z 84.3 for the analyte and m/z 288.0 to m/z 98.0 for the probe calibrator CC-6032. Calibration was performed using weighted (1/x^2^) quadratic regression of peak area. Two calibration curves for CC-4047 in PBS and lactated ringers solution were constructed using standards at concentrations of 1.28, 2.56, 5.12, 10.2, 25.6, 64.0, 160, 400, 1000, and 2000 ng/mL. A quadratic regression model with a weighting of 1/(x^2^) was used for the regression of calibration curves. Concentrations below the limit of quantitation (BLOQ) were treated as zero for calculations.

### 2) Preclinical evaluation of pomalidomide

#### Drugs

Pomalidomide (CC-4047, MW 273.25, C_13_H_11_N_3_O_4_) was obtained from Celgene Corporation.

#### Animal and housing

Female athymic mice (8-10 weeks old and weighing 20–25 g at the beginning of the study) were purchased from Harlan laboratories (Indianapolis, IN). They were housed in a temperature-controlled sterilized room (23 ± 2 °C) with a 12-h light/dark cycle and free access to food and water throughout the study. Animal use was approved by Mayo Foundation Institutional Animal Use and Care Committee and was in accordance with NIH Guide for the Care and Use of Laboratory Animals.

#### Orthotopic murine CNS lymphoma models

Two murine CNS lymphoma models were created by intracerebral injection of 2.5X10^4^ luciferase-transfected Raji (source) or 1X10^5^ luciferase-transfected OCI-LY10 (source) B lymphoma cells in athymic mice under anesthesia using a stereotactic platform. Eight-week-old athymic mice underwent minimum 7-day acclimation/quarantine prior to surgery. Surgery was performed in a laminar flow hood under sterile conditions. Tylenol 300 mg/kg PO was given for analgesia 24 hours before the surgery continuing 48 hours postoperatively. Anesthesia was achieved by inhalation of 1-2% isoflurane. After the mouse became well anesthetized, it was placed in the Kopf stereotactic instrument. A small amount of BNP antibiotic cream (a mixture of Bacitracin, Neomycin and Polymyxin) was smeared on its eyes to prevent infection and corneal damage during surgery. A strip of soft fabric was placed over the mouse’s body and tail to prevent excessive heat loss during surgery. The scalp area was cleaned with a 2% solution of Betadine and dried with cotton tipped applicator. A midline sagittal incision was made in the scalp. A small burr hole was drilled in the left skull with a surgical drill (Kopf) or a Dremel drill according to the coordinates (AP: 0.5 mm, LM: 2.5 mm) as determined by reference to the mouse brain atlas by Franklin and Paxinos [8]. The dura mater was surgically exposed, and a 10 µl -Hamilton syringe with a 26S-gauge beveled needle was lowered into the left cerebral hemisphere up to the depth of 3 mm and 5 µl of tumor cells was slowly infused (0.5µl/min). The needle was left in place for 5 minutes to prevent reflux and then was slowly removed. The skin was closed with wound clips. The mice recovered from anesthesia and surgery in a warm environment and were not returned to their cages until motor activity returned. Cages were placed on top of a heating pad to minimize the loss of body heat during the recovery. The mice were monitored post-operatively at least twice a day for 5 days or until recovery was complete.

#### Bioluminescence imaging of mouse (BLI)

After intracerebral injection of lymphoma cells, all the mice were subjected to bioluminesence imaging (BLI) twice a week starting at day-4 post-intracerebral injection to monitor the real-time in vivo tumor growth. BLI was conducted using a Xenogen Lumina optical imaging system (Caliper Life Sciences, Hopkinton, MA). Mice were anesthetized with isofluorane before intraperitoneal injections of luciferin at a dose of 150 mg/kg, providing a saturating substrate concentration for luciferase enzyme. Peak luminescent signals were recorded 10 minutes after luciferin injection. Regions of interest encompassing the intracranial area of signal were defined using Living Image software (Xenogen, Alameda, CA), and the total photons/s/steradian /cm2 was recorded.

#### In vivo preclinical evaluation of pomalidomide in murine CNS lymphoma models

Mice were imaged 4 days after intracerebral injection and were distributed among different treatment groups with equivalent average BLI signal. Real time tumor growth was monitored by BLI. Tumor growth and survival data were analyzed for statistical difference between the groups.

Mice were assigned to four experimental groups and one vehicle control group in Raji model. Mice in experimental groups received POM 0.3 mg/kg, 3 mg/kg, 10 mg/kg, or 30 mg/kg by oral gavage daily for 28 days. Control group received similar volume of vehicle by oral gavage daily for 28 days. In the OCI-LY10 model, POM 0.3 mg/kg dose level was not included. The end point of the study was survival defined as the time for the development of limb paralysis. Animals were checked at least twice per day. Animals that reached the end point were sacrificed by CO2 anesthesia.

#### Immunohistochemistry (IHC) assessment for macrophages

Paraffin sections (10 µm thick) were fixed, blocked, and immunostained with the appropriate antibody: Iba-1 (BD Biosciences) for macrophages, Ym1 (Stemcell Technologies, Vancouver, BC, Canada) as a marker for M2 polarization, iNOS (Calbiochem, Billerica, MA) as a marker for M1 polarization. Aperio ScanScope XT slide scanner and image analysis system (Aperio Spectrum, Vista, CA) were used for quantitative assessment of macrophages. Three equal-size (0.4mM^2^) fields in macrophage-dense areas were selected in the contralateral brain and the tumor for counting. Data were shown as an average of the three fields.

#### Immunofluorescence for natural killer cells

Frozen sections (10 µm thick) were permeabilized with PBS-0.2% Triton X-100 for 5 min. After blocking with PBS-5% goat serum-0.02% Triton X-100 for 45 mins at 37° C, the sections were incubated overnight at 4° C with rat monoclonal CD335 antibody (a marker for NK cells, 1:200, Biolegend, San Diego, CA). After washing, sections were incubated with the Alexa Fluor 594 goat anti-rat IgG secondary antibody (1:1000, Life Technologies, Grand Island, NY) at 37° C for 1.5 h. Finally, Vectashield H-1200 mounting medium with DAPI (Vector Laboratories, Burlingame, CA) was used to stain the nuclei. Images were obtained on a Zeiss LSM 510 META confocal microscope. Six equal-size (0.2mM^2^) fields in NK-dense areas were selected in the tumor for taking pictures. Fluorescence intensity data were generated by Zeiss LSM 510 and shown as an average of six fields.

#### Statistical analysis

One-way ANOVA was used to compare the difference between the groups at each time point. Two-way repeated measures ANOVA was used to analyse the interaction between the time and treatment. Survival analysis was performed by Kaplan Meier method. Kaplan Meier survival curves were generated using Prism4 software and the statistical difference between curves was derived with a log-rank test. P< 0.05 was considered significant.

### 3) In vitro cell culture experiments to investigate the impact of pomalidomide on the biology of macrophages

#### Cell culture and treatment

*Rajilymphoma* *cells* (ATCC), *U937 human monocyte cells* (ATCC) and YTS NK cells (a gift from Dr. Daniel D. Billadeau, Mayo Clinic, Rochester, Minnesota) were cultured at 37° C in a humidified incubator under 5% CO_2_ and 95% air in RPMI-1640 supplemented with 20% FCS and 1% penicillin-streptomycin, and 1% nonessential amino acids.

*OCI-LY10 lymphoma cells* (a gift from Arthur L. Shaffer III, National Cancer Institute, National Institute of Health, Bethesda, MD) were cultured at 37° C in a humidified incubator under 5% CO_2_ and 95% air in IMDM supplemented with 20% FCS and 1% penicillin-streptomycin. *Co-culture of lymphoma cells and U937 monocytes*: Raji and U937 monocytes were cultured at 37° C in a humidified incubator under 5% CO_2_ and 95% air in RPMI-1640 supplemented with 20% FCS and 1% penicillin-streptomycin, and 1% nonessential amino acids. OCI-LY10 and U937 monocytes were cultured at 37° C in a humidified incubator under 5% CO_2_ and 95% air in IMDM supplemented with 20% FCS and 1% penicillin-streptomycin. *Triple culture of lymphoma cells, U937 monocytes and YTS cells*: Raji, U937 and YTS cells *were* cultured at 37° C in a humidified incubator under 5% CO_2_ and 95% air in RPMI-1640 supplemented with 20% FCS and 1% penicillin-streptomycin, and 1% nonessential amino acids. OCI-LY10, U937 and YTS were cultured at 37° C in a humidified incubator under 5% CO_2_ and 95% air in IMDM supplemented with 20% FCS and 1% penicillin-streptomycin.

#### Cell Proliferation assays

Raji or OCI-LY10 lymphoma cells were seeded in triplicate at 10^4^ cells per ml per well of a 24-well tissue culture plate in 0.5 ml of media. The cells were treated with pomalidomide at 0, 0.3125, 0.625, 1.25, 2.5, 5, 10, 20, 40 or 80µg/ml. Cells were harvested and counted on the fourth day in a Coulter Particle Counter (Beckman-Coulter Corp., Brea, CA). Data were plotted over time and two-tailed student T-test was used to analyze significant difference.

#### Co-culture experiments and Fluorescence-activated cell sorting analysis

Raji or OCI-LY10 lymphoma cell were cultured alone or co-cultured with U937 monocytes or YTS NK cells, or both. They were treated with Pomalidomide (10µg/ml). After 96-hour treatment, the cells were collected and resuspended in 100 µl MACS buffer, followed by incubation with anti-CD20 (FITC, Clone: 2H7, BD Bioscience) for 30 mins in the dark. FITC Mouse lgG2b, k Isotype (BD Bioscience) was used as a control. Fluorescence-activated cell-sorting analysis was performed using Accuri C6 flow cytometer (BD Biosciences, San Jose, CA) to determine the number of CD20-expressing lymphoma cells.

#### Treatments

In experiments in which IL-4 was used to induce M2 polarization of macrophages, cells were treated with IL-4 (20ng/ml) for 48 hours followed by treatment with POM (10ug/ml) or DMSO control for 48 hours. In triple cell culture experiments with lymphoma cells, macrophages, and NK cells, the treatment was with either DMSO or POM (10ug/ml) for 48 hours.

#### Immunofluorescence for analysis of macrophage polarization in cell culture experiments

*Live cells* were fixed in 10% formalin for 30 mins at room temperature followed by permeabilization with PBS-0.2% Triton X-100 for 2 min. After blocking with PBS-5% goat serum-0.02% Triton X-100 for 30mins at 37 °C, the cells were incubated overnight at 4 °C with rat monoclonal F4/80 antibody (for primary microglia cells, 1:200 Abcam, Cambridge, MA), rat monoclonal CD11b (for U937, 1:200 Biolegend), rabbit polyclonal Ym1 antibody (for primary microglia cells and primary peritoneal macrophages, 1:200, Stemcell Technologies, Vancouver, Canada), rabbit polyclonal FXIII A antibody (for U937, 1:200, Abcam), mouse monoclonal p-STAT1 antibody (1:200, Abcam), rabbit polyclonal p-STAT6 antibody (1:200, Abcam) or rabbit iNOS antibody (1:200, Calbiochem, Billerica, MA). After washing, cells were incubated with the Alexa Fluor 594 Goat anti-Rat IgG secondary antibody (1:1000, Life Technologies, Grand Island, NY) and FITC Donkey anti-rabbit IgG secondary antibody (1:1000, Life Technologies) or FITC Goat anti-mouse lgG secondary antibody (1:1000, Sigma, St. Louis, MO) at 37 °C for 45mins. Finally, Vectashield H-1200 mounting medium with DAPI (Vector Laboratories, Burlingame, CA) was used to stain the nuclei. Images were obtained on a Zeiss LSM 510 META confocal microscope.

#### Phagocytosis assay

The impact of POM on the phagocytic activity of macrophages was assessed in primary microglial cells and human monocyte cells (U937). The cells were cultured in complete medium for 4 days. They were then harvested and resuspended in Opti-MEM medium at 10^6^ cells/ml and seeded in a 96 well plate at 100,000 viable cells/well. The experimental wells were treated with POM 3 or 10 ug/ml. After 48 hours of treatment, the culture medium was quickly replaced with 100 ul of pHrodo BioParticles suspension (Life Technologies, Grand Island, NY), and the cells were incubated in the suspension at 37° C for 2-3 hours. Following incubation, the plates were scanned at 550nm/600nm (excitation/emission) using fluorescence plate reader. The fluorescence activity reflects the phagocytic activity of the macrophages. The impact of POM treatment (% effect) was calculated as a fraction of the phagocytic activity in the positive control wells [9].

## Results

### 1) Pomalidomide has excellent CNS penetration

**Figure 1:**
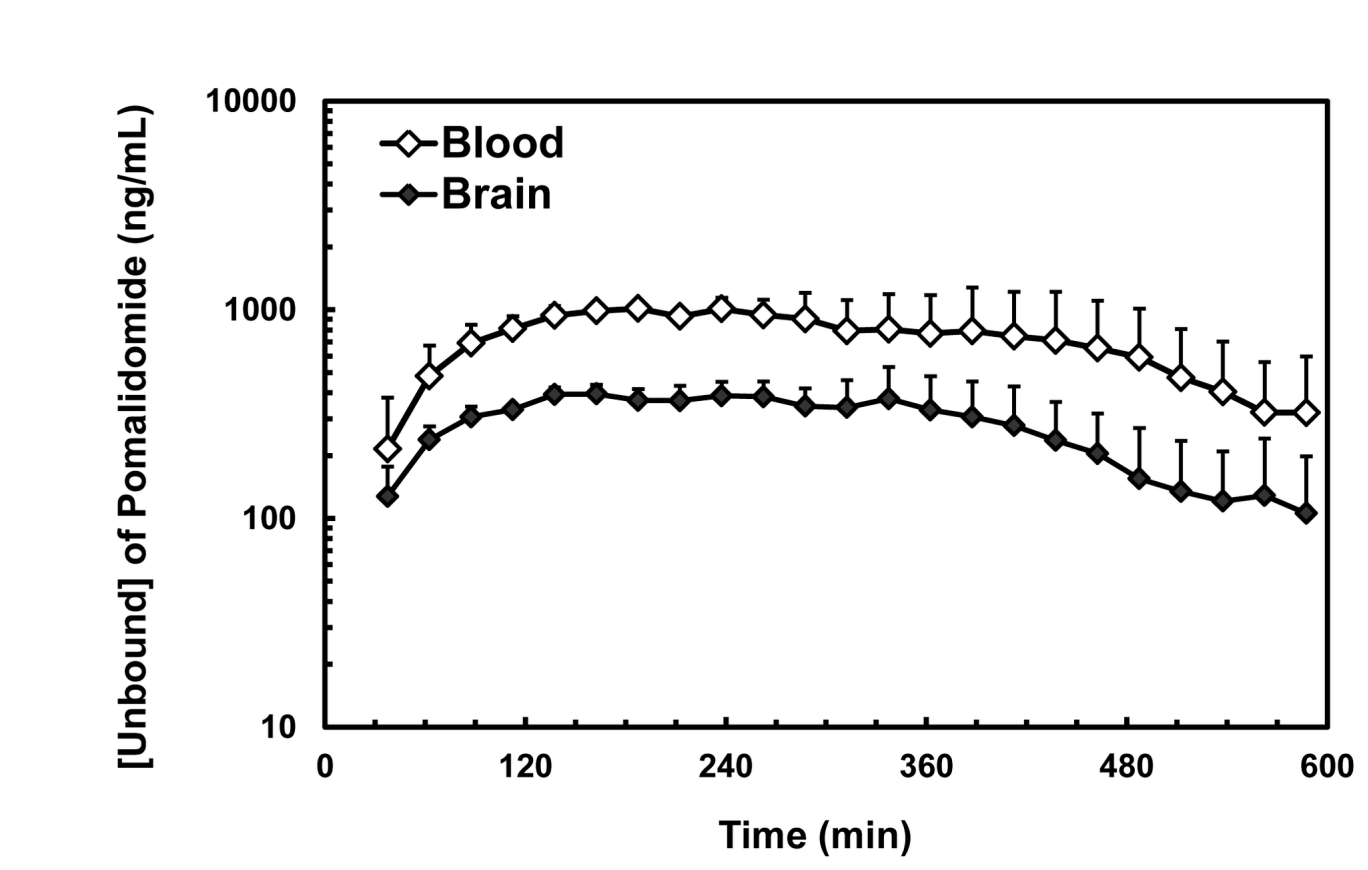
Unbound blood and brain concentration-time profiles of Pomalidomide in male CD-IGS rats following a single PO administration at 50 mg/kg (n=3).

POM was shown by a previous study to have desirable pharmacokinetic properties in the rat [3]. It had relatively slow clearance (12.3 mL/min/kg), a reasonable volume of distribution (1.75 L/kg), and an acceptable bioavailability (47.4%). Table 1 and Figure 1 summarize the brain microdialysis data. Following a 50 mg/kg PO administration of POM to rats, unbound concentrations in blood reached a C_max_ value of 1100 ± 82 ng/mL at 4.6 ± 2.4 hours, with a concomitant AUC_(0‑10)_ value of 6800 ± 2000 ng·hr/mL. Unbound POM in the brain, however, had a C_max_ value of 430 ± 63 ng/mL at 4.1 ± 1.5 hours and an AUC_(0-10)_ value of 2700 ± 740 ng·hr/mL, giving an unbound AUC_brain_ to AUC_blood_ ratio of 0.39 ± 0.03. These values are consistent with excellent blood–brain-barrier penetration. The results obtained in this study were consistent with those seen in a concurrent study looking at whole brain POM content following its oral administration to mice (data not shown).

**Table 1:** Pharmacokinetic parameters of pomalidomide in fasted male CD-IGS rats following a single IV and PO administration at 5 mg/kg and 50 mg/kg, respectively.

**IV PK Parameters**	**Mean±SD**		**PO PK Parameters**	**Mean±SD**
	**CC-4047,n=5, 5mg/kg a**			**CC-4047,n=4, 50mg/kg b**
**CL (mL/min/kg)**	**12.3±2.6**		**C_max_(ng/mL)**	**3372±758**
**Vss(L/kg)**	**1.75±0.2**		**Tmax(hr)**	**2.0±0.0**
**T1/2(hr)**	**2.44±0.8**		**AUC(0-24) (ng.hr/mL)**	**33155±8561**
**AUC(0-inf) (ng.hr/mL)**	**7016±1551**		**F(%)**	**47.4±15.2**

**Microdialysis**	**Mean±SD**			
**PO PK Parameters**	**Brain**			**Blood**
**(50mg/kg b, n=3)**				
**C_max_(ng/mL)**	**430.0±63**			**1100±82**
**Tmax(hr)**	**4.1±1.5**			**4.6±2.4**
**AUC(0-10) (ng.hr/mL)**	**2700±740**			**6800±2000**
**AUC ratio (Brain:Blood)**	**0.39±0.03**			

Mean unbound blood and brain microdialysis parameters for pomalidomide in male CD-IGS rats following a single PO administration at 50 mg/kg.

a, Solution in dimethylacetamide/PEG400/saline (10/50/40)

b, Suspension in 0.5% CMC/ 0.25% Tween 80 in water

### 2) Single agent oral pomalidomide has significant pre-clinical therapeutic activity against CNS lymphoma in murine models

**Figure 2:**
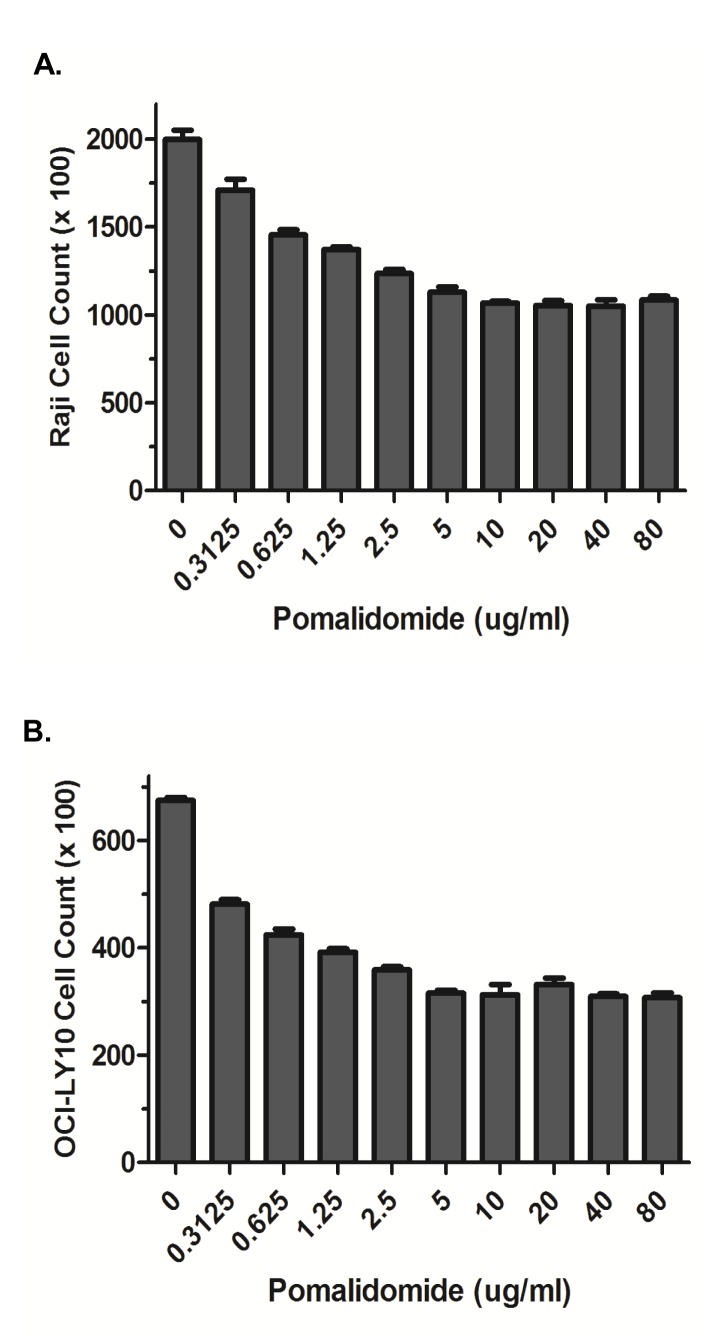
Pomalidomide has a negative impact on proliferation of Raji and OCI-LY10 lymphoma cells. Treatment with POM at various concentrations showed a modest impact on Raji (A) and OCI-LY10 (B) lymphoma cells.

**Figure 3:**
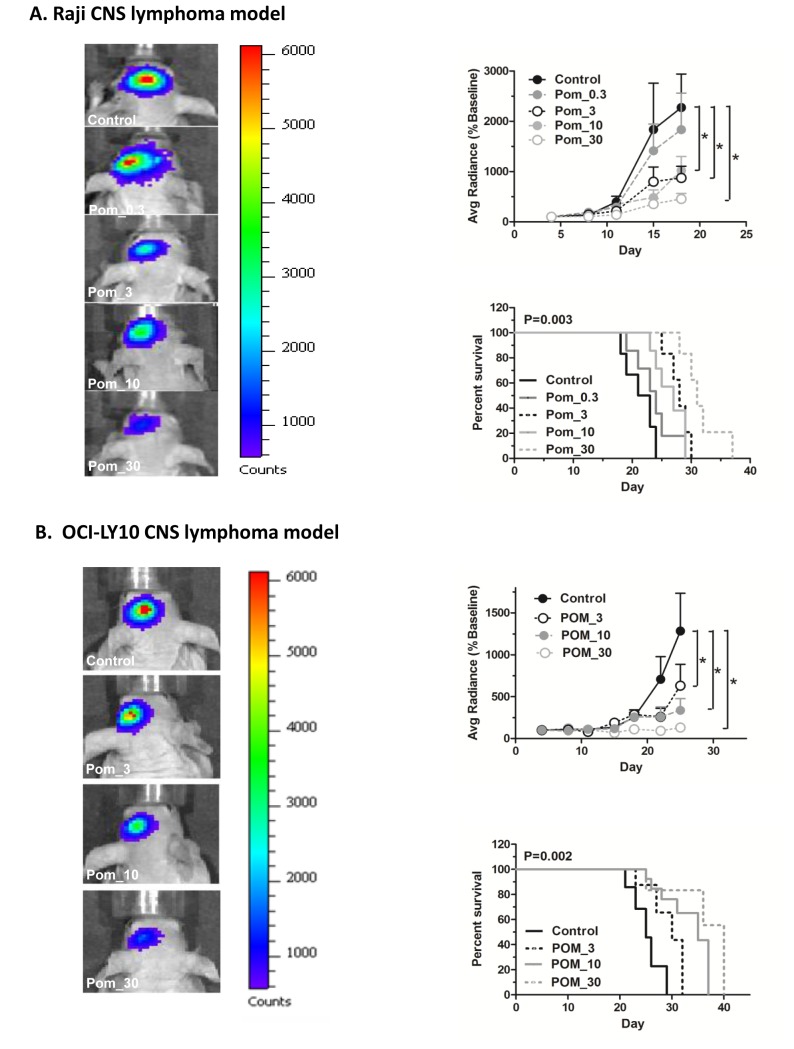
Pomalidomide (POM) showed significant pre-clinical therapeutic activity with prolongation of survival in two in vivo CNS lymphoma models. Raji model: **A-1**, **A-2** and **A-3**. OCI-LY10 model: **B-1,B-2**, and **B-3**. **A-1. and B-1**. Bioluminescence imaging of CNS lymphoma on day 18 post tumor implantation. **A-2. and B-2**. Luminescence signal of lymphoma growth post-intracerebral injection of 25,000 Raji cells or 1x10^5^ OCI-LY10 cells. The data were shown as mean ±SEM (average radiance % baseline) for n=8. In vivo tumor growth in Pom- 3mg/kg, Pom-10mg/kg and Pom-30mg/kg groups were significantly slower than that in the control group. *, P<0.05, as compared with control. **A-3. and B-3**. Kaplan-Meier analysis showed prolongation of survival with Pom_3mg/kg, Pom_10mg/kg and Pom-30mg/kg treated groups (p < 0.05, n=8).

POM showed a negative impact on proliferation of Raji and OCI-LY10 cells (Figure 2) and significant preclinical therapeutic activity against CNS lymphoma in both Raji and OCI-LY10 murine orthotopic models. The in vivo findings showed a dose-dependent therapeutic activity against CNS lymphoma with statistically significant therapeutic activity at 3 mg, 10 mg, and 30 mg/kg dose levels of POM in terms of reduction of tumor growth and prolongation of survival (Figure 3). There was a good correlation between control of tumor growth and survival prolongation. The median survival in Raji model was 31 days (30 mg/kg), 27 days (10 mg/kg), 28 days (3 mg/kg), and 24 days (0.3 mg/kg) compared to 21 days with vehicle control group (Figure 3A). The median survival in OCI-LY10 model was 40 days (30 mg/kg), 37 days (10 mg/kg), 32 days (3 mg/kg), and compared to 26 days with vehicle control group (Figure 3B). Body weight chart showed that mice in the 3mg, 10 mg, and 30mg/kg treatment groups were able to maintain their body weight better than those in control group (data not shown).

We have also tested a combination of POM and weekly dexamethasone in the Raji model, showing that the addition of dexamethasone led to further improvement in survival. Addition of oral dexamethasone 20 mg/kg weekly for 4 weeks to POM leads to improved outcomes at 10 mg and 30 mg/kg POM dose levels. The median survival was prolonged by 3 days and 5 days respectively by addition of weekly dexamethasone to POM at 10 mg and 30 mg/kg dose levels (Figure S1).

### 3) Pomalidomide has a significant impact on the tumor microenvironment in CNS lymphoma

**Figure 4:**
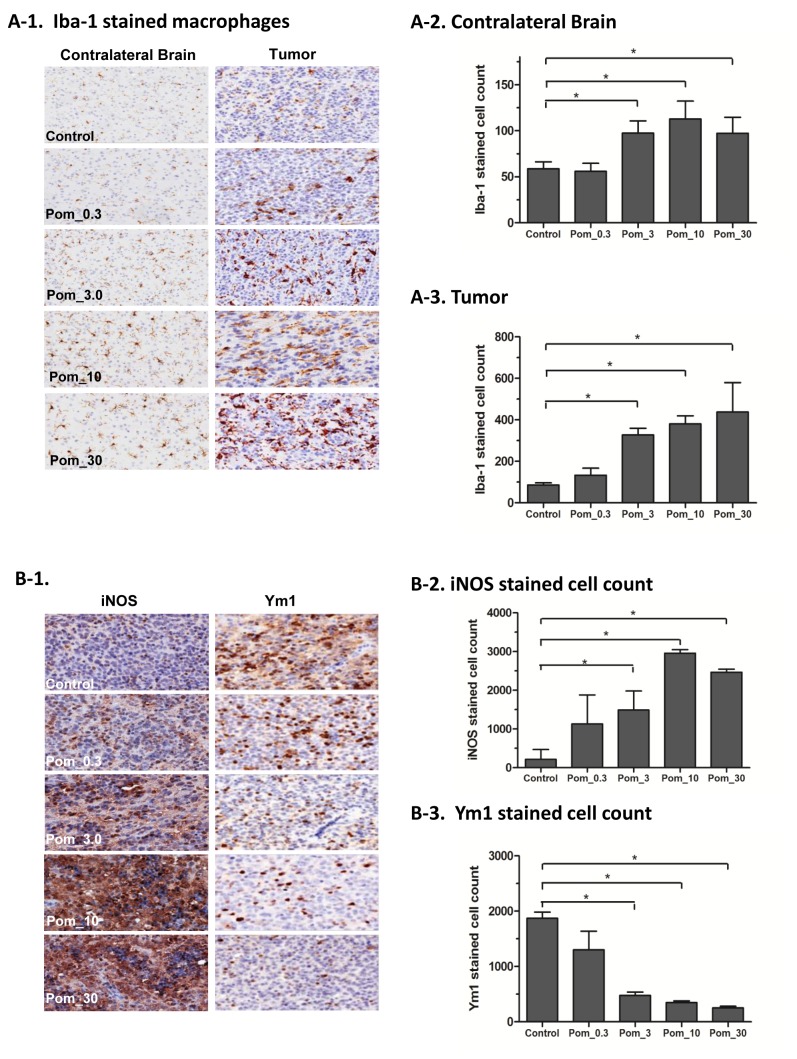
Pomalidomide (POM) had a major impact on macrophages in the CNS lymphoma microenvironment in Raji model. **A**. POM significantly increased brain macrophages. **A-1**. Iba-1 staining for brain macrophages in the contralateral brain and tumor. **A-2**. Quantitation of Iba-1 positive cells in the contra-lateral brain. **A-3**. Quantitation of Iba-1 positive cells in the tumor. **B**. POM significantly decreased Ym1-expressing cells and increased iNOS-expressing cells in the intracranial lymphoma xenografts. **B-1**. iNOS and Ym1 staining macrophages in tumor. **B-2**. Quantitation of iNOS stained cells in the tumor. **B-2**. Quantitation of Ym1 stained cells in the tumor (*, P<0.05 as compared with control group).

**Figure 5:**
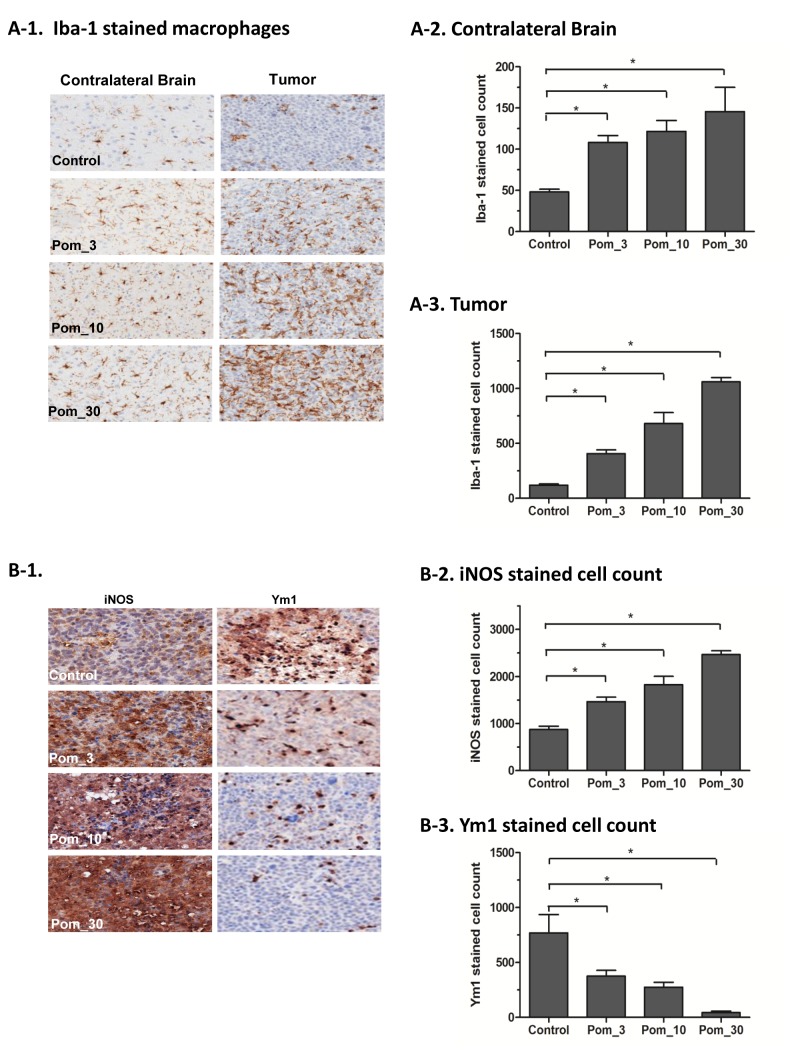
Pomalidomide (POM) had a major impact on macrophages in the CNS lymphoma microenvironment in OCI-LY10 model. **A**. POM significantly increased brain macrophages. **A-1**. Iba-1 staining for brain macrophages in the contralateral brain and tumor. **A-2**. Quantitation of Iba-1 positive cells in the contra-lateral brain. **A-3**. Quantitation of Iba-1 positive cells in the tumor. **B**. POM significantly decreased Ym1 expression and increased iNOS activity in the intracranial lymphoma xenografts. **B-1**. iNOS and Ym1 staining macrophages in tumor. **B-2**. Quantitation of iNOS stained cells in the tumor. **B-2**. Quantitation of Ym1 stained cells in the tumor (*, P<0.05 as compared with control group).

**Figure 6:**
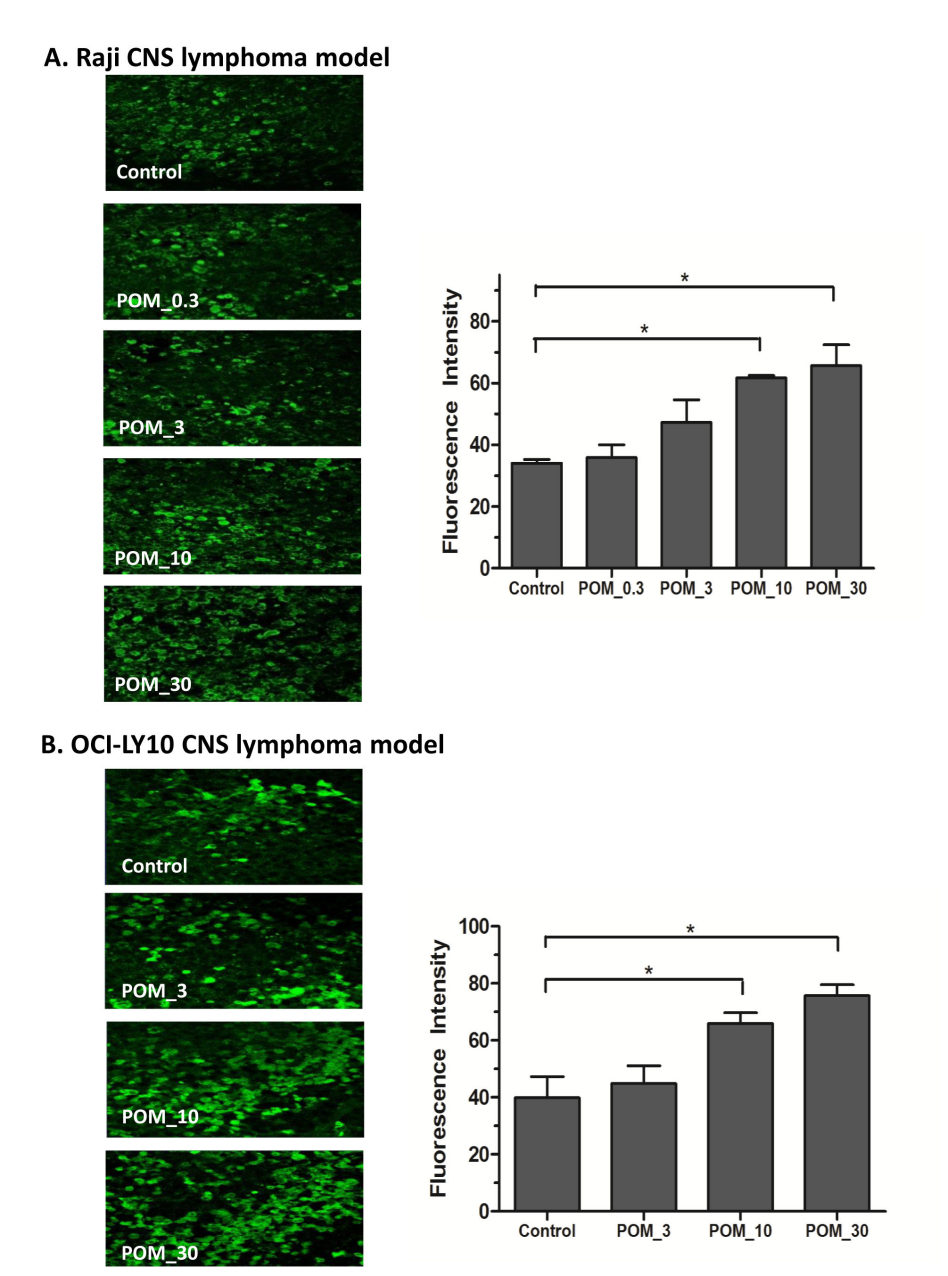
Pomalidomide (POM) significantly increased NK cells in CNS lymphoma microenvironment in the Raji and OCI-LY10 murine CNS lymphoma models. **A-1. and B-1**. POM significantly increased CD335 positive NK cells cells in CNS tumors (original magnificantion X200). **A-2. and B-2**. Fluorescence intensity of CD335 stained cells in the tumor. CD335 was used as a marker for NK cells (*, P<0.05 as compared with control group).

CNS microenvironment plays an important role in CNS lymphoma [10,11]. The immunohistochemistry studies on harvested murine brains from the preclinical evaluation showed that POM treatment significantly increased the number of macrophages by Iba-1 stain (Figures 4A and 5A). When macrophages were further studied using Ym1 as a marker of M2 polarization and iNOS as a marker of M1 polarization, POM treatment was found to be associated with a significant decrease in M2-polarized tumor associated macrophages and a significant increase in the number of M1-polarized macrophages (Figures 4B and 5B). These findings suggested that POM treatment has a significant impact on the polarization status of macrophages. Furthermore, POM treatment also led to a significant increase in the number of NK cells by CD335 stain (Figure 6). The increase in the number of macrophages and NK cells was more pronounced in the tumor compared to the cerebral hemisphere contralateral to the implantation site.

### 4) Pomalidomide has a major impact on the biology of macrophages

As a significant immunomodulatory impact was seen on macrophages in CNS lymphoma microenvironment by POM treatment in the in vivo preclinical evaluation, we proceeded with in vitro experiments to further elucidate its impact on the biology of macrophages with an emphasis on their polarization status.

**Figure 7:**
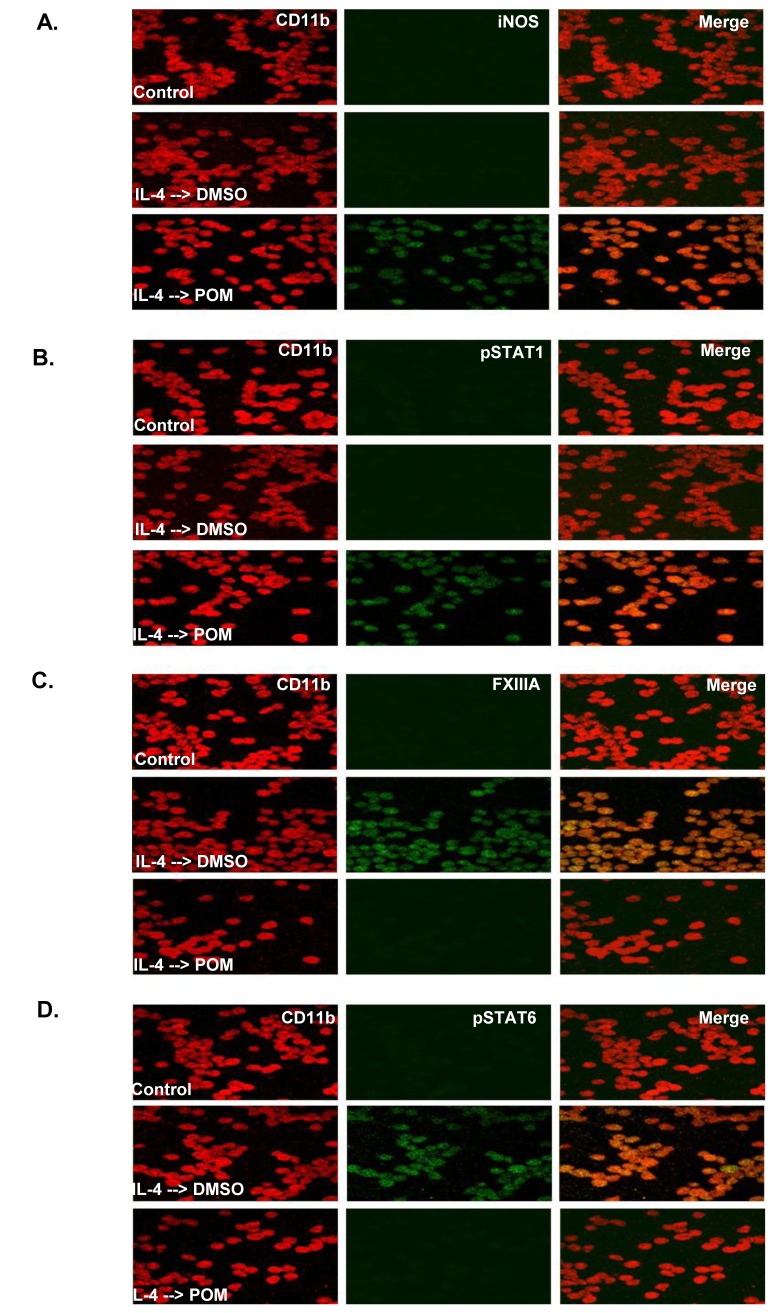
Pomalidomide converted the polarization status of IL4-treated human monocyte U937 from M2 to M1. POM converted the IL-4-induced M2 polarization of human monocytes as indicated by FXIII A and pSTAT6 expression to M1 polarization as indicated by iNOS and pSTAT1 expression. CD11b is a marker of human monocytes. Final original magnification, X 400 oil.

The impact of POM on the polarization status of macrophages was studied in various cell models including primary murine microglial cells [16], primary murine peritoneal macrophages [17], and human monocyte cell line (U937). As CNS lymphoma microenvironment is rich in IL-4 [12,13] and as IL-4 is known to induce M2 polarization of macrophages [14], IL-4 treatment followed by DMSO or POM treatment was tested on U937 (Figure 7), primary microglial cells (Figure S2), and primary peritoneal macrophages (Figure S3). These cells expressed pSTAT6 and YM1/FXIIIA [12] on treatment with IL4 followed by DMSO, indicating M2 polarization via IL4/STAT6 signalling pathway. When they were treated with IL4 followed by POM, they expressed pSTAT1 and iNOS, and did not express pSTAT6 and YM1/FXIIIA, indicating conversion of their polarization from M2 to M1 via activation of STAT1 signalling and inactivation of STAT6 signalling. As such, POM was able to reverse IL4-induced M2 polarization of macrophages and convert them into M1-polarized state.

**Figure 8:**
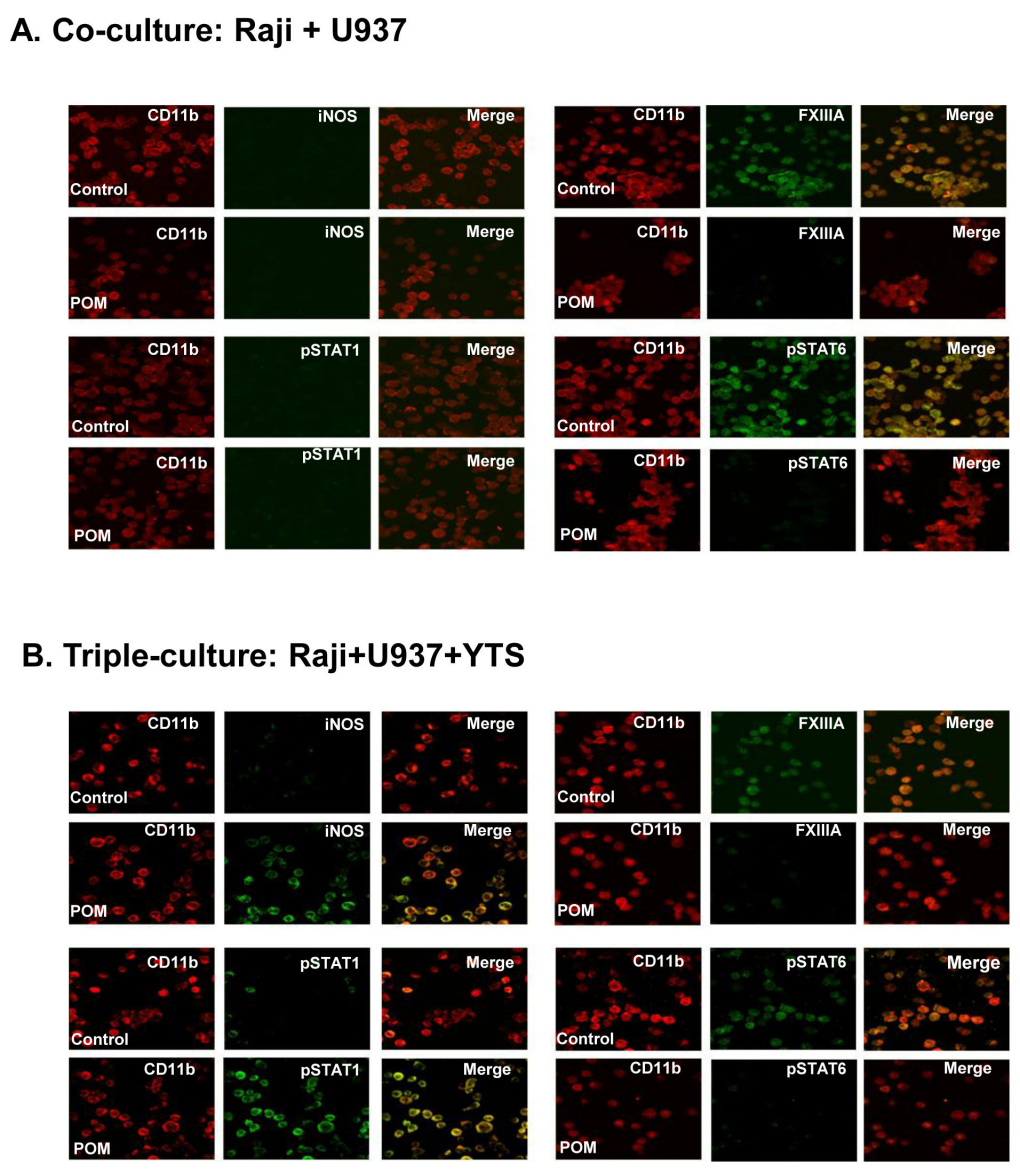
Pomalidomide converted the polarization status of lymphoma (Raji)-associated macrophages from M2 to M1 in the presence of NK cells. U937 cells became M2-polarized as indicated by FXIIIA and pSTAT6 expression, when they were cocultured with Raji lymphoma cells. The M2 polarization of U937 cells was reversed by treatment with POM (**A**). U937 cells became M2 polarized when they were cocultured with Raji lymphoma cells and YTS NK cells. When the triple culture was treated with POM treatment, M1 polarization of U937 cells was detected, as indicated by iNOS and pSTAT1 expression (**B**). CD11b is a marker of human monocytes. Final original magnification, X 400 oil.

**Figure 9:**
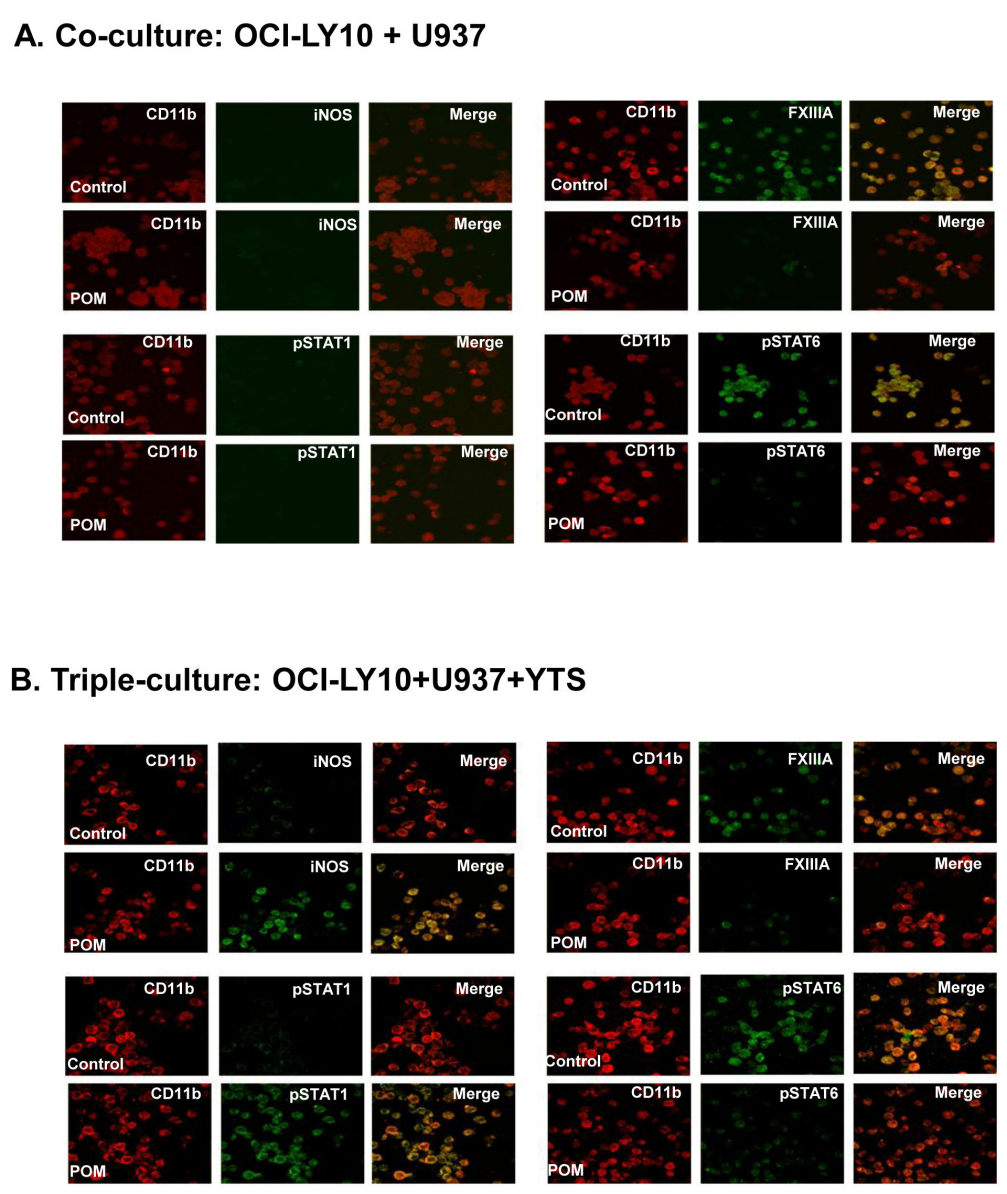
Pomalidomide converted the polarization status of lymphoma (OCI-LY10)-associated macrophages from M2 to M1 in the presence of NK cells. U937 cells became M2-polarized as indicated by FXIIIA and pSTAT6 expression, when they were cocultured with OCI-LY10 lymphoma cells. The M2 polarization of U937 cells was reversed by treatment with POM (**A**). U937 cells became M2 polarized when they were cocultured with OCI-LY10 lymphoma cells and YTS NK cells. When the triple culture was treated with POM treatment, M1 polarization of U937 cells was detected, as indicated by iNOS and pSTAT1 expression (**B**). CD11b is a marker of human monocytes. Final original magnification, X 400 oil.

To elucidate the impact of POM on lymphoma-associated macrophages, cell culture experiments were performed using three human cell lines: lymphoma cell lines (Raji or OCI-LY10) and human monocyte cell line (U937) were cultured with or without human NK cell line (YTS). When U937 cells were cocultured with lymphoma cells (Figures 8A and 9A), they became M2 polarized with expression of pSTAT6 and FXIIIA. POM treatment prevented the M2 polarization of macrophages but did not induce them into M1 polarization. In triple cell culture experiments with YTS cells, lymphoma cells, and U937 (Figures 8B and 9B), U937 cells became M2-polarized; upon POM treatment, they became M1-polarized with expression of pSTAT1 and iNOS. Based on these results, POM treatment has a major impact on the polarization status of tumor-associated macrophages, inhibiting their M2 polarization and, in the presence of NK cells, converting their polarization from M2 to M1.

To further confirm the above findings and simulate what happened in our pre-clinical in-vivo studies, we also performed cell culture experiments in which primary murine microglial cells and lymphoma cells were co-cultured with or without primary murine NK cells [18]. Essentially similar findings were observed. When primary microglial cells were co-cultured with lymphoma cells (Raji or OCI-LY10) (Figures S4A and S5A), they became M2 polarized with expression of Ym1 and pSTAT6. When the co-culture was treated with POM, M2 polarization was not seen in microglial cells: and there was also no evidence of M1 polarization. In triple cell culture experiments with primary NK cells, lymphoma cells, and primary microglial cells (Figures S4B and S5B), microglial cells became M2-polarized. With POM treatment, microglial cells became M1-polarized with expression of pSTAT1 and iNOS. As such, POM treatment prevented M2 polarization of microglial cells when they are co-cultured with lymphoma cells and converted their polarization from M2 to M1 polarization in the presence of NK cells.

To assess the functional impact of POM on macrophages, phagocytosis assay was performed.

**Figure 10:**
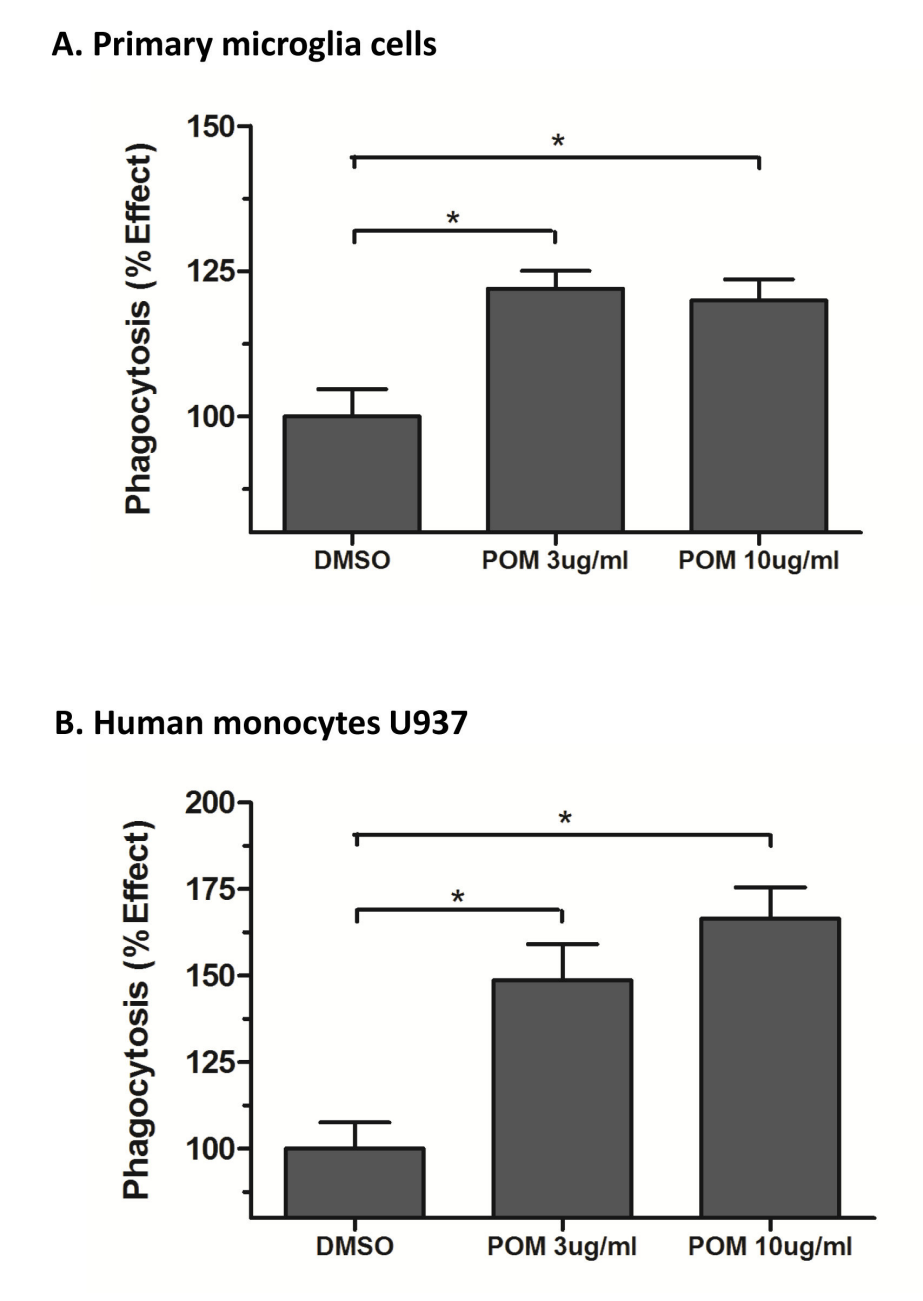
Pomalidomide significantly increased the phagocytic activity of primary murine microglia cells (A) and human monocyte U937 cells (B). (*, P<0.05 as compared with control group).

The experiment showed that treatment of primary microglial cells and human monocytes (U937) with POM significantly increased their phagocytic activity (Figure 10).

### 5) Pomalidomide has greater cytotoxicity against lymphoma cells in the presence of macropahges and NK cells

**Figure 11:**
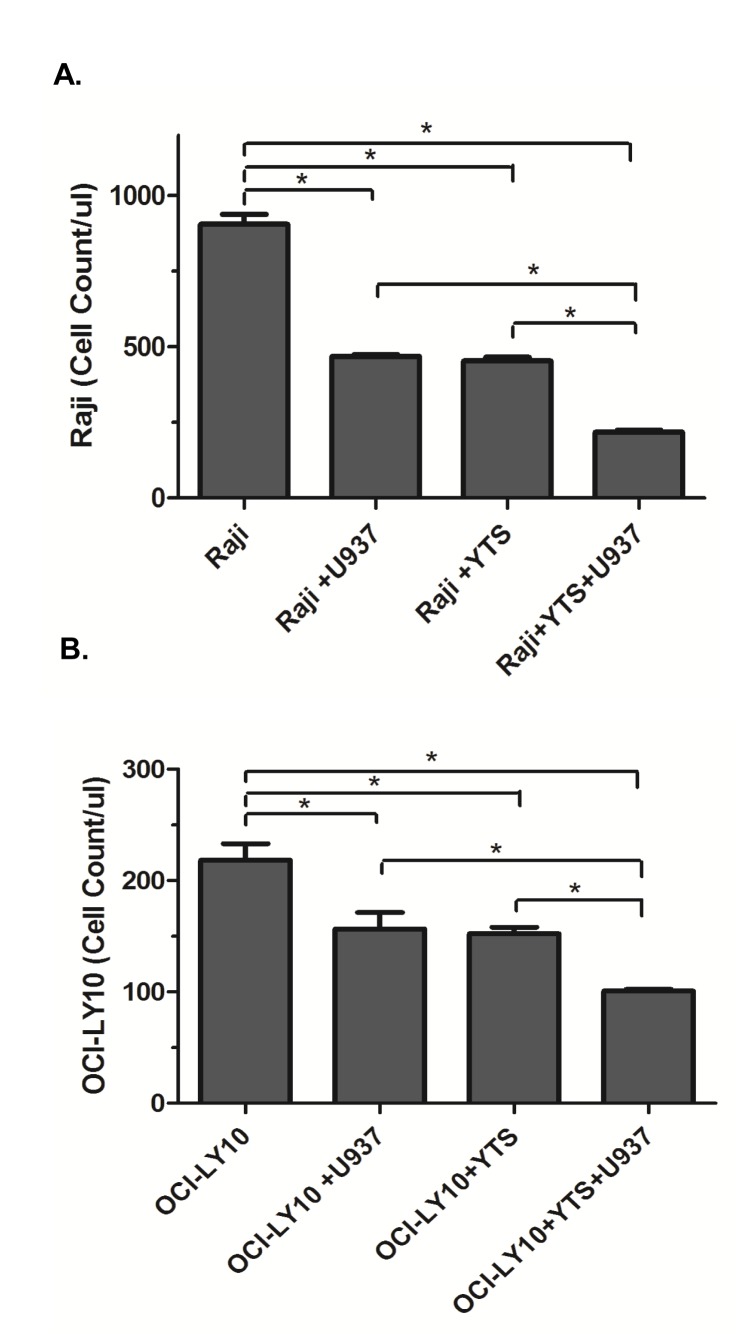
Macrophages and NK cells significantly enhanced the cytotoxic impact of pomalidomide against lymphoma cells. Cytotoxic impact of pomalidomide against Raji (A) or OCI-LY10 (B) lymphoma cells is significantly enhanced by the presence of U937 monocytes or YTS NK cells. There is a significant further improvement when both U937 and YTS cells are present.

As POM showed a major impact on tumor immune microenvironment, we hypothesized that cytotoxic impact of POM would be greater in the presence of macrophages and NK cells. Co-culture experiments showed a significant enhancement of cytotoxicity of POM against lymphoma cells by the presence of U937 monocytes or YTS NK cells. There was a statistically significant further improvement in cytotoxicity when both U937 and YTS cells are present (Figure 11).

## Discussion

In this study, we have performed a comprehensive preclinical evaluation of POM for therapeutic use against CNS lymphoma and have generated a dataset, which will help in the development of clinical trials. We showed that POM is an ideal IMiD for CNS lymphoma as our CNS pharmacokinetic studies showed that POM had excellent CNS penetration (~39%). In comparison, lenalidomide has much lower CNS penetration (~5%) [15]. As the blood brain barrier is a major obstacle in the treatment of brain tumors, POM is well suited for testing for CNS lymphoma. Preclinical evaluations in the two orthotopic murine models showed that POM had significant therapeutic activity against CNS lymphoma and a major impact on the tumor microenvironment with increased macrophages and natural killer cells. When tumor-associated macrophages were examined according to their polarization status, POM significantly decreased M2-polarized macrophages and increased M1-polarized macrophages. Further in vitro cell culture experiments showed that POM could convert the polarization status of lymphoma-associated macrophages from M2 to M1 in the presence of NK cells. Functionally, POM increased the phagocytic activity of macrophages. We further showed by co-culture experiments that cytotoxic impact of POM is significantly improved by macrophages and NK cells. We suggest that these immunomodulatory activities by POM lead to a tumor microenvironment, which is hostile to the lymphoma cells, and are likely important for its therapeutic activity against CNS lymphoma.

The impact of POM on CNS tumor microenvironment is quite relevant to the biology of primary CNS lymphoma. The CNS microenvironment appears to be essential for the survival of lymphoma cells as indicated by their confinement to the CNS. Pathway analysis of primary CNS lymphoma highlighted the important role of the CNS microenvironment [11]. Selective tropism for the CNS microenvironment appears to be essential for survival of B lymphoma cells in PCNSL [10]. As such, therapeutic agents with an impact on the CNS tumor microenvironment could have a major therapeutic effect on CNS lymphoma. POM-induced quantitative and qualitative alterations in macrophages represent important changes in CNS tumor microenvironment with therapeutic implications. POM significantly decreased tumor-promoting M2 macrophages while increasing M1 macrophages with anti-tumor property. It is an important finding as polarization status of tumor associated macrophages plays an important role in tumor biology [14]. It has been shown that macrophages in PCNSL are M2 polarized related to upregulation of IL4 in CNS lymphoma [12,13]. Our results from cell culture experiments showed that POM could convert M2 polarization of IL4-treated macrophages to M1. However, the conversion of the polarization of lymphoma-associated macrophages from M2 to M1 by POM requires the presence of NK cells. These findings indicated the importance of interactions between macrophages and NK cells in the regulation of polarization status of macrophages. These interactions may be mediated via cytokines secreted by the NK cells under the influence of POM. At the signalling level, it appears that the conversion of M2 to M1 polarization status of macrophages by POM is mediated via inactivation of STAT6 signalling and activation of STAT1 signalling.

Based on our data, POM appears to mediate therapeutic activity against CNS lymphoma by direct impact on the tumor cells as well as by inducing alterations in the tumor microenvironment. Our findings are consistent with another study which showed that POM alone had modest cytotoxic effect on Raji lymphoma cells and that cytotoxic effect was significantly better in the presence of peripheral blood mononuclear cells [3]. As such, it is likely that the therapeutic activity of POM is predominantly related to its impact on the tumor immune microenvironment. The main weakness of our study is that our immunosuppressed models only allowed the study of macrophages and NK cells but did not allow the study of T lymphocytes. We plan to perform a more comprehensive evaluation of immunomodulatory impact of POM in the future, employing an immunocompetent syngeneic murine CNS lymphoma model, and studies to elucidate the mechanism of action underlying the immunomodulatory activities of POM.

Based on our comprehensive preclinical dataset, POM holds great promise for CNS lymphoma. Moreover, immunomodulatory therapy represents a new therapeutic modality for CNS lymphoma with a major impact on the tumor microenvironment. We have developed a phase I clinical trial for patients with relapsed/refractory CNS and ocular lymphoma. Our data showing a major impact of POM on tumor-associated macrophages provides the rationale for combining POM with immunotherapeutic agents such as rituximab. Based on its major impact on the CNS tumor microenvironment, POM should also be tested for CNS prophylaxis in systemic lymphomas with high CNS risk and for use in consolidation and maintenance therapies after induction therapy for CNS lymphoma. More studies are necessary to identify the role of POM in the treatment of CNS lymphoma.

## Supporting Information

File S1 **Cell culture and treatment.**
(DOCX)

Figure S1 **Addition of weekly Dexamethasone (Dex) to Pomalidomide (POM) led to further improvement in survival in Raji CNS lymphoma model.**
**A**. **C. E**. Luminescence signal of lymphoma growth on day 4, 8, 11, 15 and 18 post-intracerebral injection of 25,000 Raji cells. The data were shown as mean ±SEM (average radiance) for n=8. *,P<0.05 as compared with control; **, P<0.05, as compared with control and Dex; ***, P<0.05, as compared with control, Dex alone and Pom alone treatment group. B. D. F. Kaplan-Meier analysis shows prolongation of survival with Dex+Pom_10mg/kg and Dex+Pom-30mg/kg treated groups as compared with Pom alone treated groups (p < 0.05, n=8).
(TIF)

Figure S2 **Pomalidomide converted the polarization status of IL4-treated primary murine microglia cells from M2 to M1.**
POM converted the IL-4-induced M2 polarization of microglia cells as indicated by FXIII A and pSTAT6 expression to M1 polarization as indicated by iNOS and pSTAT1 expression. CD11b is a marker of human monocytes. Final original magnification, X 400 oil.
(TIF)

Figure S3 **Pomalidomide converted the polarization status of IL4-treated primary murine peritoneal macrophages from M2 to M1.**
POM converted the IL-4-induced M2 polarization of macrophages as indicated by FXIII A and pSTAT6 expression to M1 polarization as indicated by iNOS and pSTAT1 expression. CD11b is a marker of human monocytes. Final original magnification, X 400 oil.
(TIF)

Figure S4 **Pomalidomide converted the polarization status of lymphoma (Raji)-associated primary murine microglia cells from M2 to M1 in the presence of primary murine NK cells.**
Microglia cells became M2-polarized as indicated by FXIIIA and pSTAT6 expression, when they were cocultured with Raji lymphoma cells. Their M2 polarization was reversed by treatment with POM (**A**). They became M2 polarized when they were cocultured with Raji lymphoma cells and primary NK cells. When the triple culture was treated with POM treatment, M1 polarization of microglia cells was detected, as indicated by iNOS and pSTAT1 expression (**B**). F4/80 is a marker of murine microglia cells. Final original magnification, X 400 oil.
(TIF)

Figure S5 **Pomalidomide converted the polarization status of lymphoma (OCI-LY10)-associated primary murine microglia cells from M2 to M1 in the presence of primary murine NK cells.**
Microglia cells became M2-polarized as indicated by FXIIIA and pSTAT6 expression, when they were cocultured with OCI-LY10 lymphoma cells. Their M2 polarization was reversed by treatment with POM (**A**). They became M2 polarized when they were cocultured with OCI-LY10 lymphoma cells and primary NK cells. When the triple culture was treated with POM treatment, M1 polarization of microglia cells was detected, as indicated by iNOS and pSTAT1 expression (**B**). F4/80 is a marker of murine microglia cells. Final original magnification, X 400 oil.
(TIF)
