# Supplementary material for: Pomalidomide Shows Significant Therapeutic Activity against CNS Lymphoma with a Major Impact on the Tumor Microenvironment in Murine Models
Source: PLoS One. 2013 Aug 5;8(8):e71754. doi: 10.1371/journal.pone.0071754 (PMC3734315; doi:10.1371/journal.pone.0071754)
Supplement: File S1 — Cell culture and treatment. (DOCX) [file pone.0071754.s001.docx]

**Cell culture and treatment**

*Primary microglia cells were isolated from BALB/c mice brain according to the method described by Moussaud and Draheim [*[*16*](#_ENREF_16)*]*. *Primary peritoneal macrophages* were isolated from BALB/c mice according to the method described by Davies and Gordon [[17](#_ENREF_17)]. *Primary NK cells* were isolated from BALB/c mice spleen according to the method described by Reichlin, Iizuka and Yokoyama [[18](#_ENREF_18)]. They were cultured at 37°C in a humidified incubator under 5% CO_2_ and 95% air in DMEM/F12 supplemented with 10% FCS and 1% penicillin-streptomycin.

*Co-culture of lymphoma cells and primary microglia cells*: Raji and primary microglia cells were cultured at 37°C in a humidified incubator under 5% CO_2_ and 95% air in DMEM/F12 supplemented with 10% FCS and 1% penicillin-streptomycin. OCI-LY10 and primary microglia cells were cultured at 37°C in a humidified incubator under 5% CO_2_ and 95% air in DMEM/F12 supplemented with 10% FCS and 1% penicillin-streptomycin.

*Triple culture of lymphoma cells, primary NK cells and primary microglia cells*: Raji /OCI-LY10, primary NK cells and primary microglia cells were cultured at 37°C in a humidified incubator under 5% CO_2_ and 95% air in DMEM/F12 supplemented with 10% FCS and 1% penicillin-streptomycin.
