# Supplementary figures and images for: Pomalidomide Shows Significant Therapeutic Activity against CNS Lymphoma with a Major Impact on the Tumor Microenvironment in Murine Models
Source: PLoS One. 2013 Aug 5;8(8):e71754. doi: 10.1371/journal.pone.0071754 (PMC3734315; doi:10.1371/journal.pone.0071754)

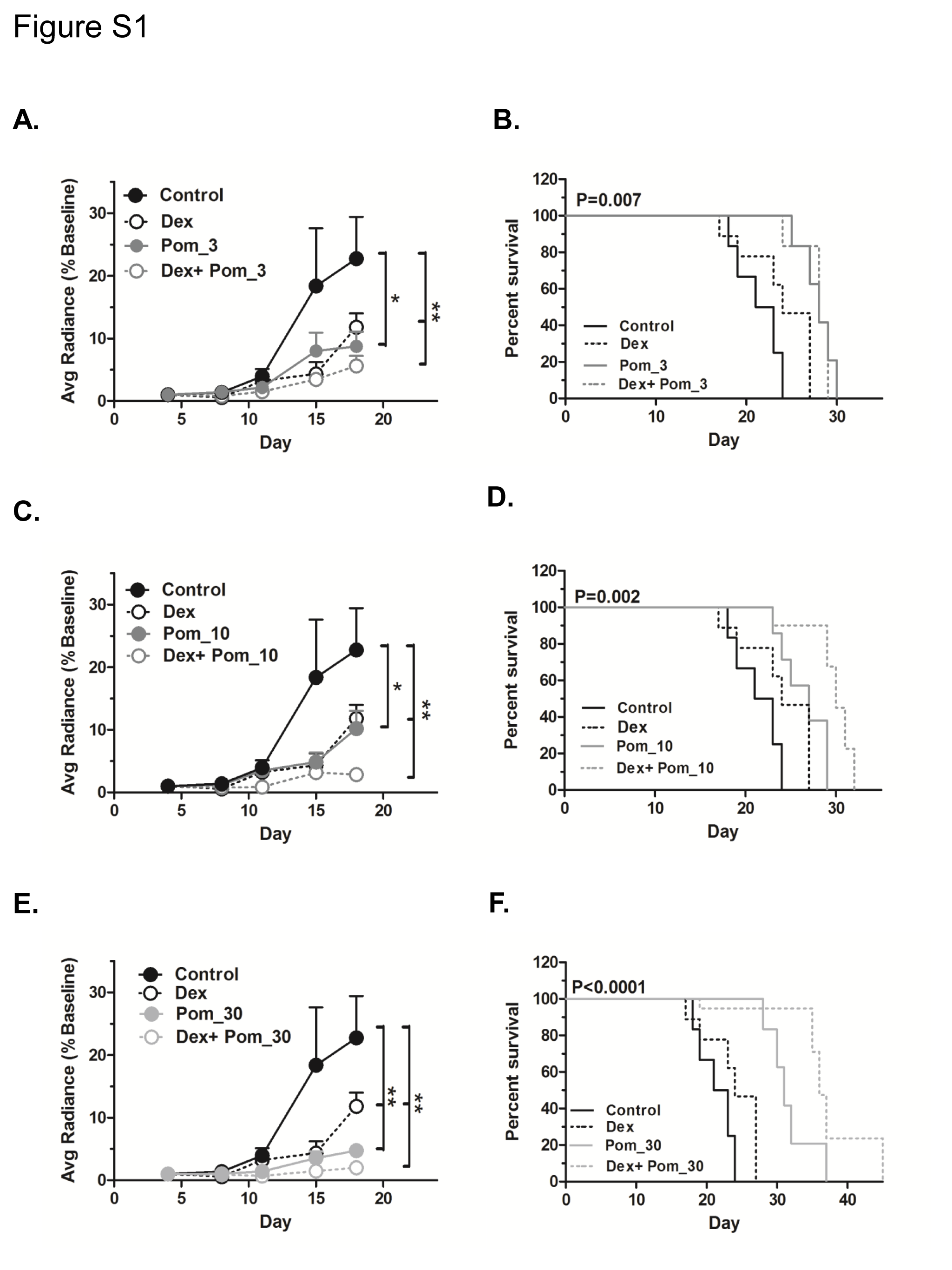

Supplement: Figure S1 — Addition of weekly Dexamethasone (Dex) to Pomalidomide (POM) led to further improvement in survival in Raji CNS lymphoma model. A. C. E. Luminescence signal of lymphoma growth on day 4, 8, 11, 15 and 18 post-intracerebral injection of 25,000 Raji cells. The data were shown as mean ±SEM (average radiance) for n=8. *,P<0.05 as compared with control; **, P<0.05, as compared with control and Dex; ***, P<0.05, as compared with control, Dex alone and Pom alone treatment group. B. D. F. Kaplan-Meier analysis shows prolongation of survival with Dex+Pom_10mg/kg and Dex+Pom-30mg/kg treated groups as compared with Pom alone treated groups (p < 0.05, n=8). (TIF) [file pone.0071754.s002.tif]

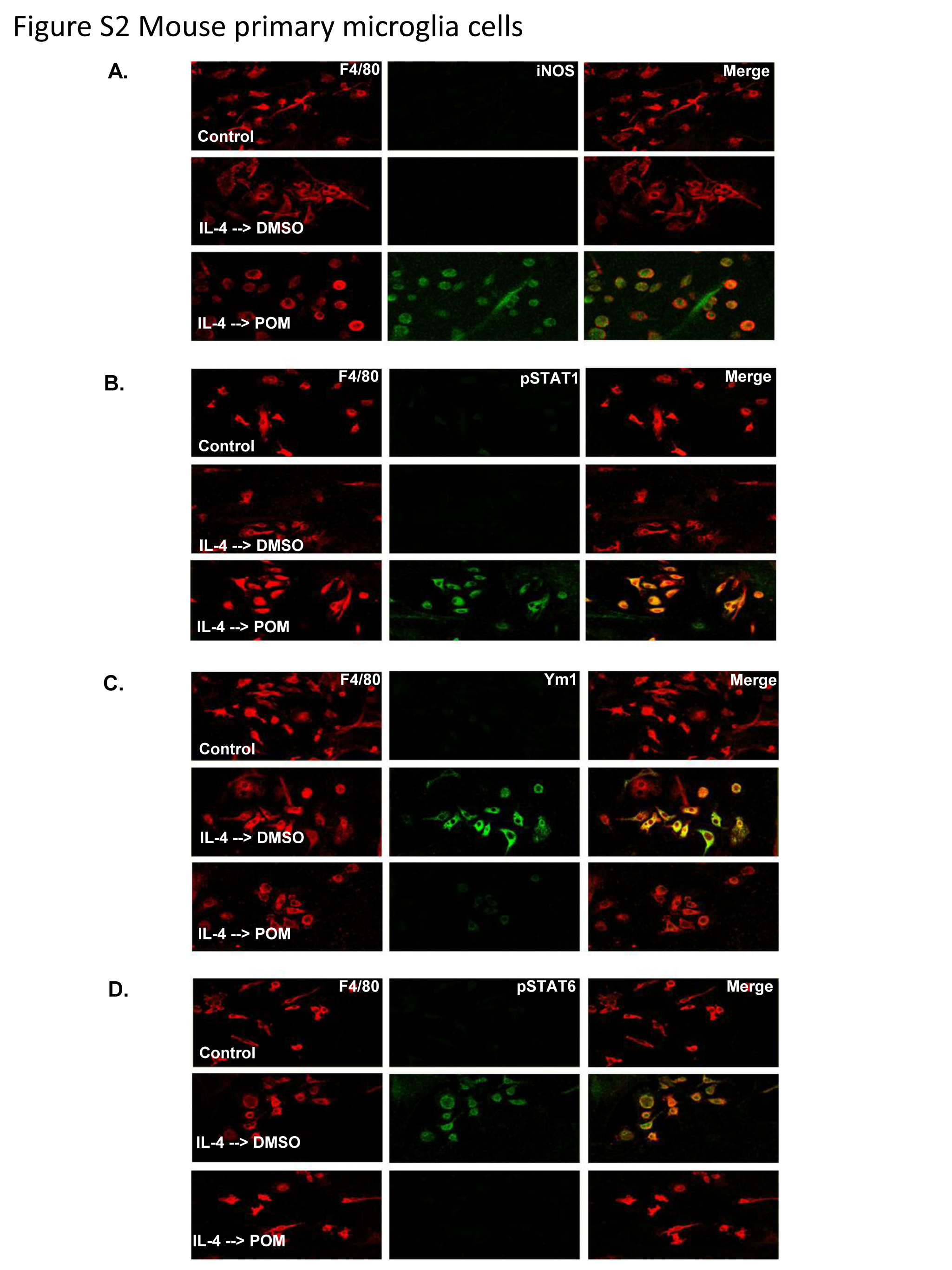

Supplement: Figure S2 — Pomalidomide converted the polarization status of IL4-treated primary murine microglia cells from M2 to M1. POM converted the IL-4-induced M2 polarization of microglia cells as indicated by FXIII A and pSTAT6 expression to M1 polarization as indicated by iNOS and pSTAT1 expression. CD11b is a marker of human monocytes. Final original magnification, X 400 oil. (TIF) [file pone.0071754.s003.tif]

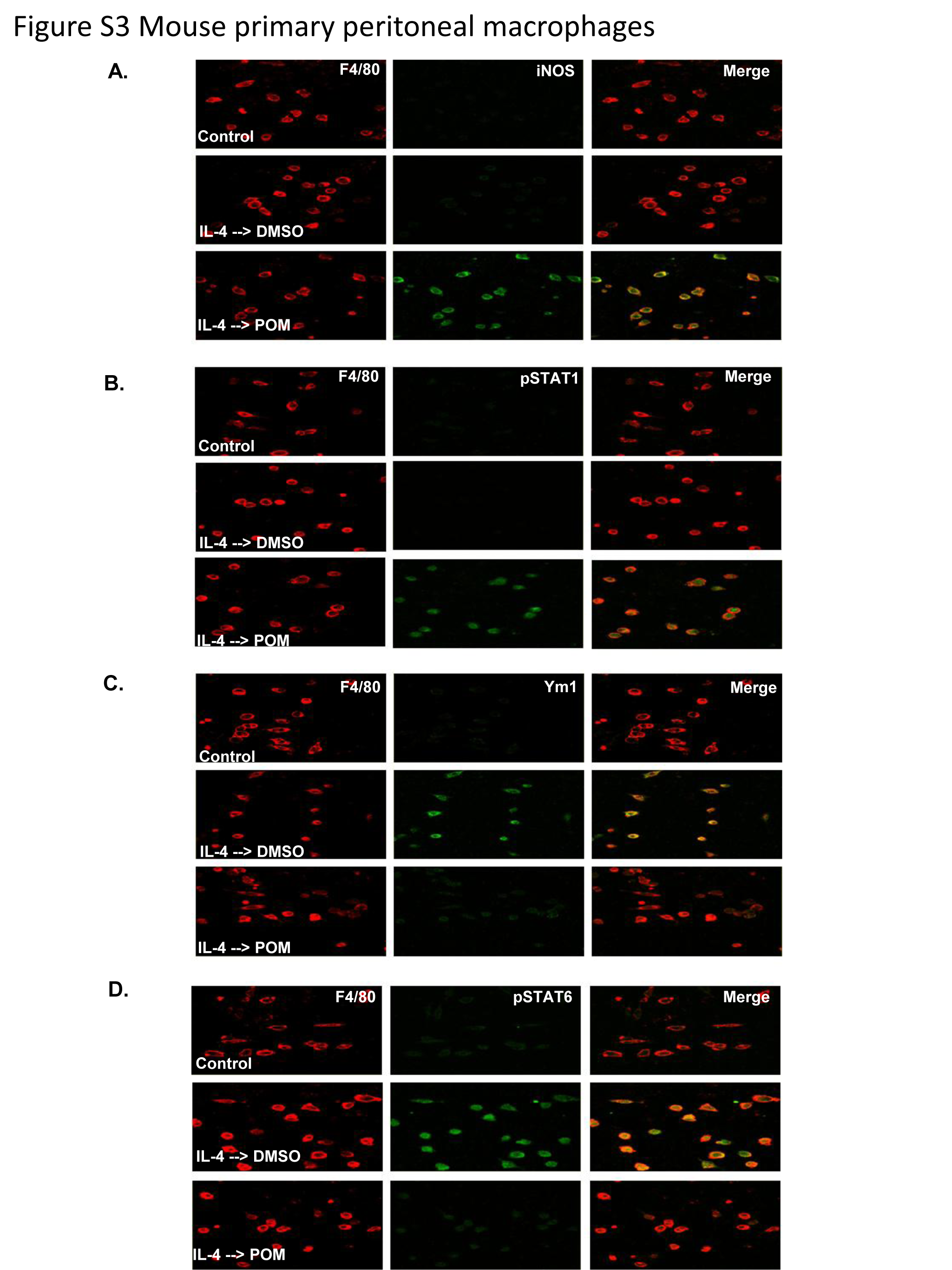

Supplement: Figure S3 — Pomalidomide converted the polarization status of IL4-treated primary murine peritoneal macrophages from M2 to M1. POM converted the IL-4-induced M2 polarization of macrophages as indicated by FXIII A and pSTAT6 expression to M1 polarization as indicated by iNOS and pSTAT1 expression. CD11b is a marker of human monocytes. Final original magnification, X 400 oil. (TIF) [file pone.0071754.s004.tif]

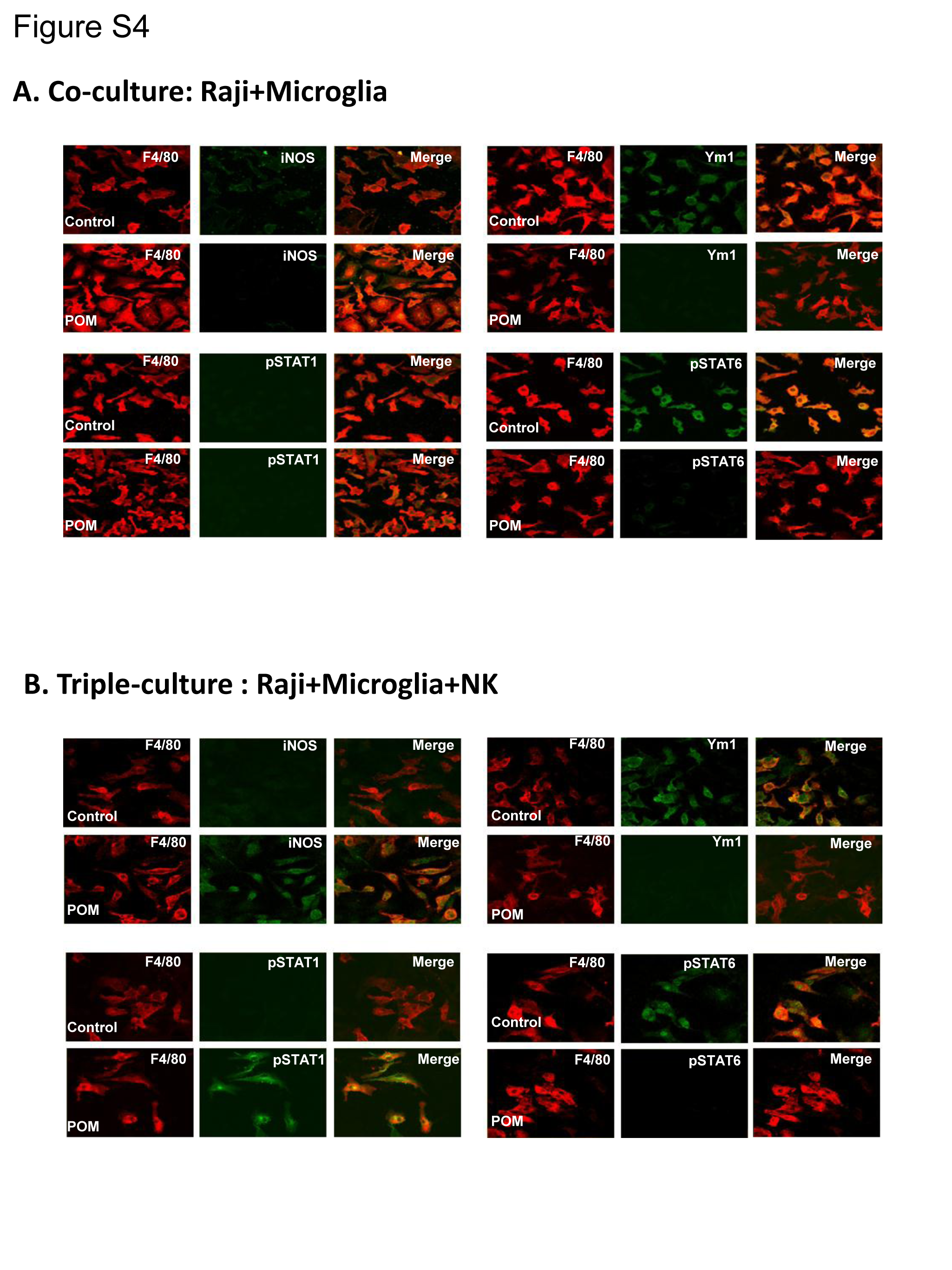

Supplement: Figure S4 — Pomalidomide converted the polarization status of lymphoma (Raji)-associated primary murine microglia cells from M2 to M1 in the presence of primary murine NK cells. Microglia cells became M2-polarized as indicated by FXIIIA and pSTAT6 expression, when they were cocultured with Raji lymphoma cells. Their M2 polarization was reversed by treatment with POM (A). They became M2 polarized when they were cocultured with Raji lymphoma cells and primary NK cells. When the triple culture was treated with POM treatment, M1 polarization of microglia cells was detected, as indicated by iNOS and pSTAT1 expression (B). F4/80 is a marker of murine microglia cells. Final original magnification, X 400 oil. (TIF) [file pone.0071754.s005.tif]

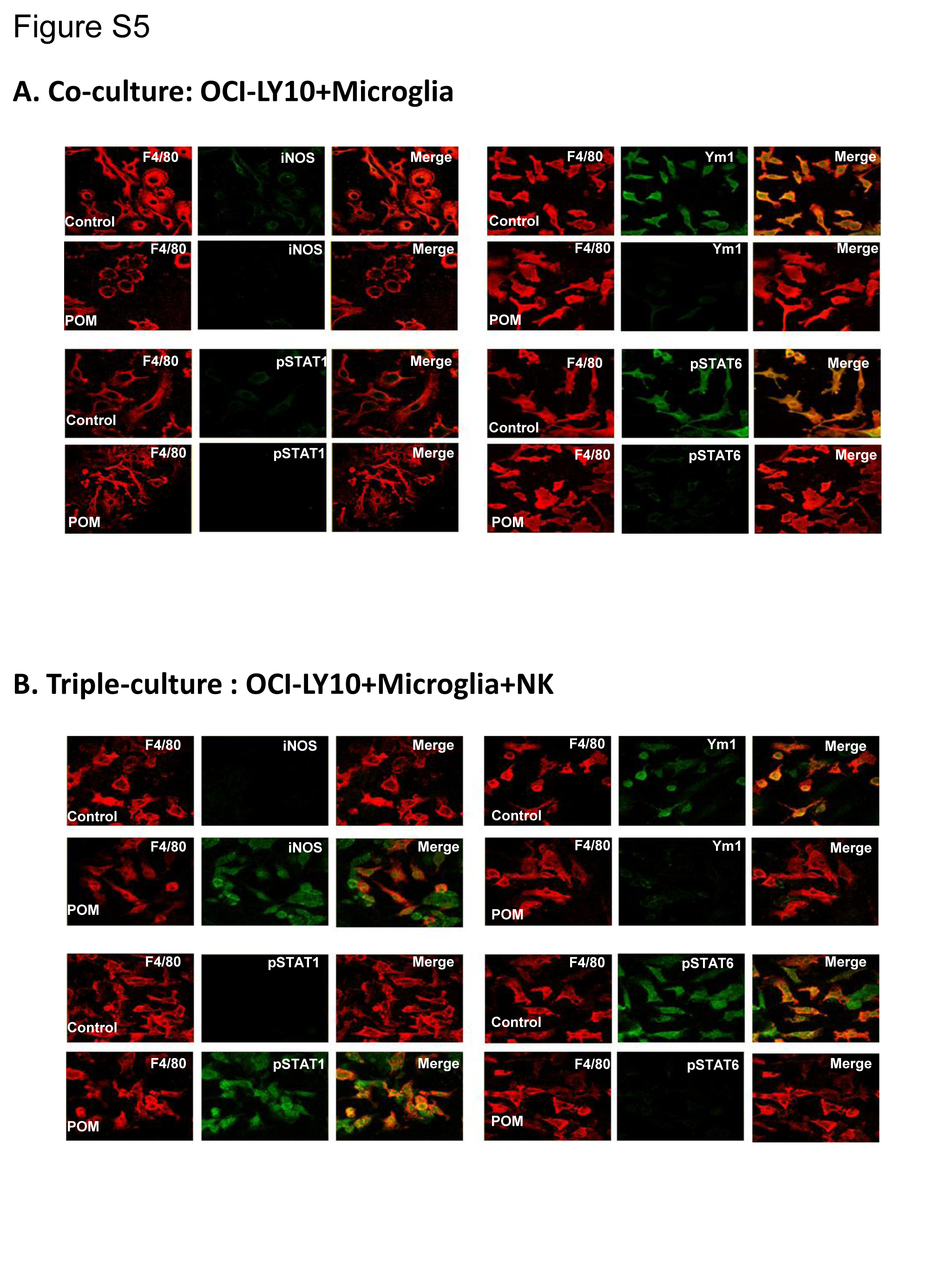

Supplement: Figure S5 — Pomalidomide converted the polarization status of lymphoma (OCI-LY10)-associated primary murine microglia cells from M2 to M1 in the presence of primary murine NK cells. Microglia cells became M2-polarized as indicated by FXIIIA and pSTAT6 expression, when they were cocultured with OCI-LY10 lymphoma cells. Their M2 polarization was reversed by treatment with POM (A). They became M2 polarized when they were cocultured with OCI-LY10 lymphoma cells and primary NK cells. When the triple culture was treated with POM treatment, M1 polarization of microglia cells was detected, as indicated by iNOS and pSTAT1 expression (B). F4/80 is a marker of murine microglia cells. Final original magnification, X 400 oil. (TIF) [file pone.0071754.s006.tif]
